# Fiberoptic Reintubation in an Unanticipated Difficult Airway: A Case Report

**DOI:** 10.1155/cria/9811616

**Published:** 2026-07-29

**Authors:** Alireza Nikzad-Jamnani, Seyyed Alireza Seyyed Siamdoust, Seyyed Javad Boskabadi, Sima Ramezaninejad, Saeed Kargar-Soleimanabad

**Affiliations:** ^1^ Department of Anesthesiology and Critical Care Medicine, Faculty of Medicine, Mazandaran University of Medical Sciences, Sari, Iran, mazums.ac.ir; ^2^ Department of Anesthesiology, Faculty of Medicine, Iran University of Medical Sciences, Tehran, Iran, iums.ac.ir; ^3^ Department of Clinical Pharmacy, Faculty of Pharmacy, Mazandaran University of Medical Sciences, Sari, Iran, mazums.ac.ir; ^4^ Department of Clinical Pharmacy, Faculty of Pharmacy, Babol University of Medical Sciences, Babol, Iran, mubabol.ac.ir; ^5^ Student Research Committee, Faculty of Medicine, Mazandaran University of Medical Sciences, Sari, Iran, mazums.ac.ir

**Keywords:** difficult airway, difficult intubation, flexible fiberoptic laryngoscopy, reconstructive neck surgery

## Abstract

**Background:**

Although direct laryngoscopy may seem the most reasonable modality for previously intubated patients, in the setting of anatomical distortion secondary to reconstructive neck surgery, flexible fiberpostoptic laryngoscopy (FFL) should be considered for patients who may require postoperative emergency reintubation.

**Case Presentation:**

A 51‐year‐old woman with squamous cell carcinoma of the submental triangle and larynx was referred for free flap surgery. For postoperative care, she was admitted to the ICU. After 18 h, the patient developed respiratory distress and confusion. We decided to perform awake tracheal intubation for her. After receiving a sufficient dose of fentanyl and dexmedetomidine, intubation with direct laryngoscopy was attempted, but it failed. Fortunately, the patient was intubated using an FFL available in the ICU. After 48 h, she was extubated and transferred to the surgery ward.

**Conclusion:**

Our experience suggests that, for patients who are undergoing free flap surgery in regions that jeopardize the airway, if intubation is needed (especially shortly after surgery), difficult airway management devices such as FFL should be readily accessible alongside an expert anesthesiologist.

## 1. Introduction

Endotracheal intubation and direct laryngoscopy (DL) are routine processes for patients requiring mechanical ventilation [1]. Difficult airway (sometimes difficult intubation is used instead) involves difficult laryngoscopy or difficult endotracheal tube placement [2]. Difficult intubation can occur because of anatomical abnormalities, including subglottic stenosis, or conditions such as obesity, trauma, older age, airway inflammation, tracheal tumor, and severely limited jaw protrusion [3, 4]. A difficult airway can often not be anticipated on a preintubation assessment [5]. A difficult airway may lead to the “cannot ventilate, cannot intubate” situation. It is a significant cause of anesthesia‐associated death [6].

Unsuccessful intubation is responsible for about 30% of all deaths attributed to anesthesia [7]. Therefore, successful management and establishing a detailed plan for endotracheal intubation in patients with a difficult airway are necessary to secure the airway under the given circumstances and reduce complications. All clinicians must be familiar with the equipment and techniques necessary for successful intubation. In the Closed Claims Analysis performed by the American Society of Anesthesiology, failed intubation remains a major cause of morbidity and mortality [8]. The options for a difficult airway include the intubating laryngeal mask airway, fiberoptic bronchoscope (FOB), intubating stylet, articulating laryngoscope, video laryngoscope, and cricothyroidotomy. The transmission of a visual image through a flexible fiberoptic bundle was first reported in 1954 [9]. Currently, fiberoptic intubation (FOI) with a flexible FOB has become a mainstay of difficult airway management in awake, sedated, and anesthetized patients. FOI remains the accepted standard in elective airway management of the awake, spontaneously breathing patient with an anticipated difficult airway [10].

Although DL may seem the most reasonable modality for intubated patients, in case of anatomical distortion secondary to reconstructive neck surgery, other protocols should be considered. We present a case of difficult intubation in a patient with head and neck cancer.

## 2. Case Presentation

A 51‐year‐old woman affected by maxillofacial deformities following squamous cell carcinoma of the submental triangle and larynx presented to the emergency department. Considering aesthetic restoration and better function of free flap surgery compared to other surgical methods, it was a candidate for this procedure.

Her vital signs immediately after entering the emergency department were as follows: blood pressure: 130/80 mmHg, temperature: 37°C, heart rate: 83 beats/min, and respiratory rate: 18 breaths per minute.

The patient′s medical history included hypertension, alongside squamous cell carcinoma. After taking a complete history, the patient was admitted to the oncology ward. One day after hospitalization for free flap surgery, anesthesia was induced with sevoflurane and remifentanil, and then intubation was performed under DL. The free flap was raised (transferred) from the Transverse Rectus Abdominis Muscle (TRAM Flap) accordingly. At the end of the lengthy surgery, the patient was extubated with good breathing indices (normal Tidal volume) and a reversal of the muscle relaxant. For post‐operative care, she was admitted to the ICU.

Following 18 h in the ICU, the patient developed respiratory distress and confusion, necessitating emergent tracheal intubation and ventilatory support. After receiving a sufficient dose of fentanyl and dexmedetomidine, we achieved a state of conscious sedation (Ramsay Sedation Scale 2), where the patient remained cooperative with stable spontaneous ventilation. Awake intubation with DL was then attempted using a Macintosh blade size 3. However, the attempt failed as we encountered a Cormack–Lehane Grade IV view.

The primary anatomical barriers were significant soft‐tissue edema and distorted airway landmarks resulting from the recent extensive reconstructive surgery and the presence of the free flap. No significant trismus was noted, but the surgical alterations limited the effective alignment of the airway axes. The patient remained hemodynamically stable without significant desaturation or other major vital sign changes. Flexible fiberoptic laryngoscopy (FFL) was subsequently used successfully to secure the airway. After 48 h, she was extubated and transferred to the surgery ward. The current study has been reported in line with the SCARE criteria [11].

## 3. Discussion

FOI is often the best choice when there is no surgical indication for a tracheostomy [12, 13]. Tracheostomy was described over 4000 years ago as a surgical procedure. It is characterized by the use of a dilatation kit and endoscopic control during the procedure. Several contraindications for tracheostomy have been reported, including the pediatric population, an unsafe airway, an emergency, the presence of a median cervical lesion, nonpalpable cricoid cartilage, and coagulopathy [12].

Reconstructive procedures, especially those involving free flaps, lead to significant postoperative tissue edema and altered lymphatic drainage in the oropharyngeal region. Postoperative tissue edema, inflammation, flap bulk, hematoma formation, and distortion of upper airway anatomy can interfere with the alignment of the oral, pharyngeal, and laryngeal axes, reducing glottic visualization during DL [14, 15]. These changes often obscure the visualization of the glottis and prevent the dynamic displacement of the tongue and soft tissues required for a clear line of sight during DL. Consequently, the anatomical distortion in these patients necessitates advanced airway management tools, making FFL a superior alternative to conventional methods in such high‐risk scenarios. Previous studies have shown that patients undergoing head and neck oncologic or reconstructive procedures have a significantly increased risk of difficult intubation and peri‐intubation airway complications [15].

In emergencies, the need for ventilation and airway management is important, and performing a tracheostomy is difficult at this time. Lack of information on detailed medical history without enough time to get a complete thorough physical examination before needing to act, short preparation time, and lack of patient compliance are some factors that increase the difficulty of emergency airway management [16]. More recently, indirect laryngoscopy has revolutionized difficult airway management with its ability to ‘look around the corner’ during intubation, obviating the need for alignment of the oral, pharyngeal, and laryngeal axes [17]. The intubating FOB was first described in 1967, and some authors still consider it to be the gold standard in anticipated difficult airway management [18]. Flexible laryngoscopy is an awake procedure that is simple, safe, and easy to use in the clinic, unlike DL, which requires GA with attendant risk. It plays a vital role in difficult airway management, as it enables the anesthetist to have a safety management plan and a rescue strategy for anticipated difficult airways. In a case report by Pang et al., where a large tracheal tumor covering 90% of the lumen caused difficulties in endotracheal intubation via DL, fiberoptic‐assisted endotracheal intubation with a 5 mm ID ETT tube reduced the time to intubation to 3 min and prevented any peri‐procedural complications [19]. Bawa et al. presented successful airway management by awake nasal FOI in a 65‐year‐old male who was found to have compressive symptoms due to thyroid swelling in addition to a supraglottic obstructive mass [20]. FOI has improved the success rate in difficult intubation manifolds, according to recent studies.

The present case report highlights that several physiological and anatomical factors following reconstructive neck surgery resulted in a poor laryngoscopy view (Cormack–Lehane Grade IV), highlighting the limitations of DL in such patients and the importance of immediate access to fiberoptic airway equipment.

## 4. Conclusion

Although DL may seem the most reasonable modality for previously intubated patients, in cases of anatomical distortion following reconstructive neck surgery in patients who may need postoperative emergency reintubation, FFL should be considered. FOI is a well‐established and versatile tool for airway management in patients with known or suspected difficult airways, as well as a rescue technique. Awake FOI with adequate sedation can greatly facilitate safe endotracheal intubation in head and neck injuries.

## Author Contributions

Alireza Nikzad‐Jamnani and Seyyed Alireza Seyyed Siamdoust were involved in the interpretation and collection of data. Seyyed Javad Boskabadi, Sima Ramezaninejad, and Saeed Kargar‐Soleimanabad were involved in writing and editing the manuscript.

## Funding

This research received no external funding.

## Disclosure

All the authors reviewed the paper and approved the final version of the manuscript

## Consent

Written informed consent was obtained from the patient for publication of this case report and accompanying images. A copy of the written consent is available for review by the Editor‐in‐Chief of this journal on request.

## Conflicts of Interest

The authors declare no conflicts of interest.

## Data Availability

The data that support the findings of this study are available from the corresponding author upon reasonable request, subject to institutional and ethical approval.
